# Circulating miRNA Profiling in Plasma Samples of Ovarian Cancer Patients

**DOI:** 10.3390/ijms20184533

**Published:** 2019-09-13

**Authors:** András Penyige, Éva Márton, Beáta Soltész, Melinda Szilágyi-Bónizs, Róbert Póka, János Lukács, Lajos Széles, Bálint Nagy

**Affiliations:** 1 Department of Human Genetics, Faculty of Medicine, Faculty of Pharmacy, University of Debrecen, Debrecen 4032, Hungary; 2 Department of Human Genetics, Faculty of Medicine, University of Debrecen, Debrecen 4032, Hungary; m.eva92@gmail.com (É.M.); soltesz.beata@med.unideb.hu (B.S.); szilagyi.melinda@med.unideb.hu (M.S.-B.); nagy.balint@med.unideb.hu (B.N.); 3 Department of Obstetrics and Gynecology, Faculty of Medicine, University of Debrecen, Debrecen 4032, Hungary; pokar@med.unideb.hu (R.P.);; 4 Department of Biochemistry and Molecular Biology, Faculty of Medicine, University of Debrecen, Debrecen 4032, Hungary; szelesl@med.unideb.hu

**Keywords:** ovarian cancer, circulating miRNA, blood plasma, NanoString, network analysis, biomarker

## Abstract

Ovarian cancer is one of the most common cancer types in women characterized by a high mortality rate due to lack of early diagnosis. Circulating miRNAs besides being important regulators of cancer development could be potential biomarkers to aid diagnosis. We performed the circulating miRNA expression analysis in plasma samples obtained from ovarian cancer patients stratified into FIGO I, FIGO III, and FIGO IV stages and from healthy females using the NanoString quantitative assay. Forty-five miRNAs were differentially expressed, out of these 17 miRNAs showed significantly different expression between controls and patients, 28 were expressed only in patients, among them 19 were expressed only in FIGO I patients. Differentially expressed miRNAs were ranked by the network-based analysis to assess their importance. Target genes of the differentially expressed miRNAs were identified then functional annotation of the target genes by the GO and KEGG-based enrichment analysis was carried out. A general and an ovary-specific protein–protein interaction network was constructed from target genes. Results of our network and the functional enrichment analysis suggest that besides HSP90AA1, MYC, SP1, BRCA1, RB1, CFTR, STAT3, E2F1, ERBB2, EZH2, and MET genes, additional genes which are enriched in cell cycle regulation, FOXO, TP53, PI-3AKT, AMPK, TGFβ, ERBB signaling pathways and in the regulation of gene expression, proliferation, cellular response to hypoxia, and negative regulation of the apoptotic process, the GO terms have central importance in ovarian cancer development. The aberrantly expressed miRNAs might be considered as potential biomarkers for the diagnosis of ovarian cancer after validation of these results in a larger cohort of ovarian cancer patients.

## 1. Introduction

Ovarian cancer (OC) is one of the most frequent gynecological malignancy among women. Despite recent progress that has been made in the treatment of patients, OC is still characterized by a high mortality rate. It is the fifth leading cause of cancer-induced death in females in the world; the overall five-year survival rate is still only 15%–30% according to recent reports [1,2,3]. The high mortality is at least partially due to the difficulties of early detection of the tumor. The routine diagnostic procedures (pelvic examination, transvaginal ultrasonography, CA125 antigen measurement) carried out in clinics are not suitable for early diagnosis [4,5,6,7]. During recent years several groups investigated the possibility of using circulating miRNAs as candidate biomarkers for diagnosis in several different human cancers, among them in OC. In addition to their diagnostic potential, the involvement of circulating miRNAs in tumor formation might be an equally important topic to study [8,9,10]. 

MiRNAs are endogenously expressed, short (~22 nucleotides) non-coding single-stranded regulatory RNA molecules, known to interfere with the translation of the mRNA coded by the target genes. MiRNAs are negative regulators of gene expression, upon their sequence-specific binding to the 3′-UTR region of mRNAs, they can repress the translation of target mRNA or facilitate its cleavage and elimination [11]. MiRNAs are promiscuous post-transcriptional regulators, due to their short “seed” sequence, a miRNA can interact with a large number of mRNAs, that way miRNAs are involved in the regulation of almost all important cellular, developmental, and pathological processes [12]. It is well established that miRNAs are present and can be reliably detected in blood plasma since these circulating miRNAs are very stable, which is the result of their packaging into vesicles or interaction with proteins that protect miRNAs from RNase digestion. These features could make them not only ideal candidates for non-invasive plasma-based biomarkers but also regulatory factors contributing to tumor progression [7,13]. 

Circulating miRNAs could be released from cells through an active, regulated secretion process packaged into exosomes or microvesicles [14]. Exosomes were shown to be involved in intercellular communication since they carry various bioactive molecules, among them miRNAs [7,15]. Exosomes can interact, fuse with the membrane of cells, and deliver their cargo to recipient target cells and thus modify their gene expression pattern [16,17,18,19,20,21]. Exosomes are considered as part of the tumor environment playing important roles in pre-metastatic environment formation, tumor progression, epithelial-to-mesenchymal transition, and the modulation of immune regulation [14]. MiRNAs could be secreted from cells via binding to protein containing complexes like AGO2 or HDL. Although the functional role of circulating miRNAs is still largely unknown, it is known that the dysregulation of miRNA expression and the presence of circulating miRNAs have been linked to the formation of cancer among them OC, as well [7,10,22]. It was shown that miRNAs could be oncomiRs or tumor suppressors, however this categorization is not straightforward, due to their extensive palette of target genes the same miRNA could play opposing roles in different processes [23].

Several differentially expressed miRNAs were found in the plasma of OC patients, like members of the miR-200 family, miR-141, miR-125b, miR-222-3p or Let-7 among others [14,24,25]. Despite the large number of reports no consensus regarding the circulating miRNA signature has been suggested so far, which would unambiguously distinguish OC patients from healthy individuals and an explanation of their potential biological significance in OC is still not clarified completely. The fact that ovarian cancer cells are rarely disseminated through the vasculature makes the interpretation of the pathophysiological role of circulating miRNAs difficult [1,15,26].

The aim of this study was to analyze the circulating miRNA expression profile in serous epithelial OC patients and compare it to that of healthy individuals in order to contribute to the growing body of data available to establish a useful miRNA set for OC diagnosis obtained by the non-invasive liquid biopsy. We attempted to identify the miRNA expression profile, which is specific for tumor samples. We have also aimed to investigate the possible involvement of circulating miRNAs in OC development by analyzing the biological importance of miRNA targets and by the functional enrichment profile of their target gene set.

## 2. Results

### 2.1. Expresion Levels of miRNAs in Plasma Samples of Ovarian Cancer Patients and Healthy Controls

In order to compare the plasma miRNA expression profile between healthy females and OC patients, 18 patients and six healthy control females were recruited in the study. The age range of the patients was from 43 to 75 years with a mean age of 58.3 year, the same values for controls were 59.2 ranging from 62 to 71. The clinical stage and tumor subtype were established for patients and based on their immunohistochemical analysis all patients belong to the serous epithelial ovarian cancer group. Patients were stratified into FIGO I, FIGO III, and FIGO IV stages according to the recommendations of the International Federation of Gynecology and Obstetrics. Six patients from each group were included in the study together with six healthy females and their plasma samples were collected for RNA extraction and miRNA analysis. The demographic and clinical data of the patients are shown in Supplementary Table S1. Plasma miRNA profiles were determined by using the nCounter Human v3 miRNA Panel of NanosString nCounter Analysis System (NanoString Technologies). Normalized counts differed considerably for miRNAs and individuals, however, most miRNA counts were low, especially in controls, with few exceptions. Hsa-miR-451a had the highest count with group mean values ranging from 1217 up to 3392, followed by hsa-miR-4454 and hsa-miR-499a-5p (group mean values: Between 36 and 89; 56 and 123, respectively). None of these highly expressed miRNAs showed significantly different expression among the cohorts. It might be important to note that the miRNA counts were the highest in samples of FIGO I stage patients.

Altogether 45 out of the 798 unique miRNAs showed significant differences in counts between tumor and normal plasma samples. Among them, 17 miRNAs showed significantly different expressions between controls and patients (Group 1 miRNAs). The rest, 28 miRNAs were expressed only in patients, 19 out of 28 were found only in FIGO I stage samples (Group 2 miRNAs) and nine were present in all FIGO stages (Group 3 miRNAs) (Table 1).

**Figure 1:**
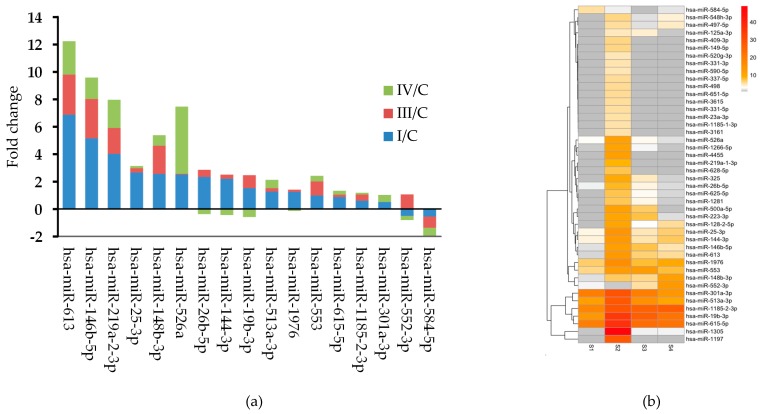
(**a**) Stacked bar chart showing the fold changes between the stage specific expression of the significantly differentially expressed miRNAs. (**b**) Heat map of the differentially expressed circulating miRNAs. The expression cluster shows upregulated miRNAs in deeper color according to the scale on the right of the figure. S1 represent controls, S2, S3, and S4 represent the FIGO I, FIGO III, and FIGO IV samples, respectively.

In Group 1 samples, the fold changes between the stage specific mean expression values were calculated for all miRNAs showing that 16 miRNAs were upregulated and hsa-miR-584-5p was downregulated in this group (Figure 1a). Typically, the largest fold change was found between the control and FIGO I stage expression values for most of the significantly differentially expressed miRNAs. Hierarchical clustering of the differentially expressed circulating miRNAs showed a distinct expression pattern between the three groups (Figure 1b).

### 2.2. Validation of Differentially Expressed Group 1 miRNAs by RT-qPCR

**Figure 2:**
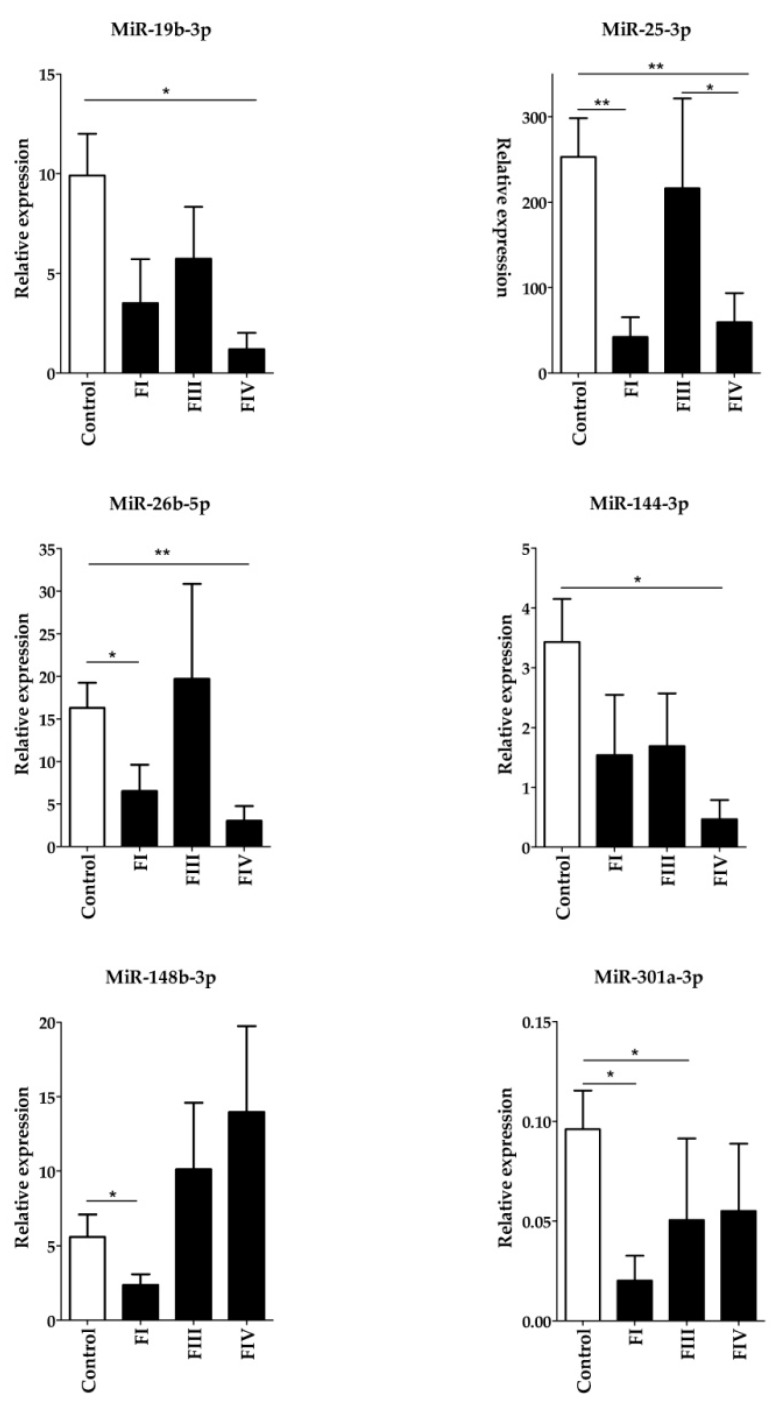
Validation of six randomly chosen significantly differentially expressed Group 1 miRNAs using the RT-qPCR measurement. Expression levels of plasma miRNAs were compared between ovarian cancer patients and control females. Total miRNA was isolated from plasma samples and the amounts of mature hsa-miRNAs was determined by the miScript PCR System. The expression of PCR products was normalized to hsa-miR-103-3p and relative miRNA expression levels were determined by the 2^-∆Ct^ method. All measurements were done in triplicate. Data distribution was analyzed by the Kruskal–Wallis one-way ANOVA test with Dunn’s *post-hoc* analysis, p-values shown in the figure are as follows: *: *P* < 0.05; **: *P* < 0.01.

To validate our findings, six Group 1 miRNAs (hsa-miR-19b-3p, hsa-miR-25-3p, hsa-miR-26b-5p, hsa-miR-144-3p, hsa-miR-148b-3p, and hsa-miR-301a-3p) were randomly chosen together with hsa-miR-197, which did not show differential expression between the groups. The relative expression of these miRNAs was determined by RT-qPCR measurements using hsa-miR-103-3p as the reference miRNA. Expression of all six Group 1 miRNAs was significantly upregulated compared with those in the control samples, while the expression of hsa-miR-197 did not show significant difference between the groups (the Kruskal–Wallis p-values were the following: hsa-miR-19b-3p: 0.03572; hsa-miR-25-3p: 0.0071; hsa-miR-26b-5p: 0.0096; hsa-miR-144-3p: 0.01828; hsa-miR-148b-3p: 0.00014; hsa-miR-301a-3p: 0.01078; hsa-miR-197: 0,741) (Figure 2). Considering the perfect positive agreement between the results obtained with the nCounter Human v3 miRNA Panel and PCR measurements, the RT-qPCR method validated our results. 

### 2.3. MIRNA Ranking, Target Gene Prediction and Analysis

To study the possible functional role of circulating miRNAs, the differentially expressed miRNAs and their targets were analyzed. The fact that a single miRNA has several target mRNAs and translation of a mRNA can be regulated by several miRNAs warrants a network-based analysis of the miRNA function. First, the miRNet tool was used to predict miRNA targets and interactions between miRNAs and targets in order to determine the importance of miRNAs in the network. The interaction networks were constructed separately for the three groups of miRNAs. Table 2 shows the lists of miRNAs ranked by their degree centrality value (betweenness centrality provided the same ranking) that represent the importance of given miRNAs in the interacting network. (The complete networks are shown in Supplementary Figure S1a–c)

**Figure 3:**
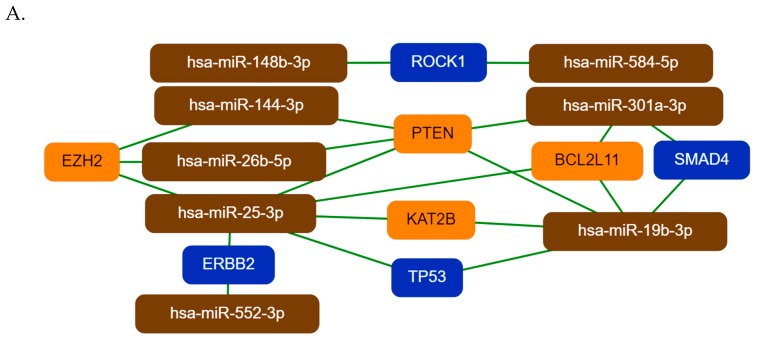

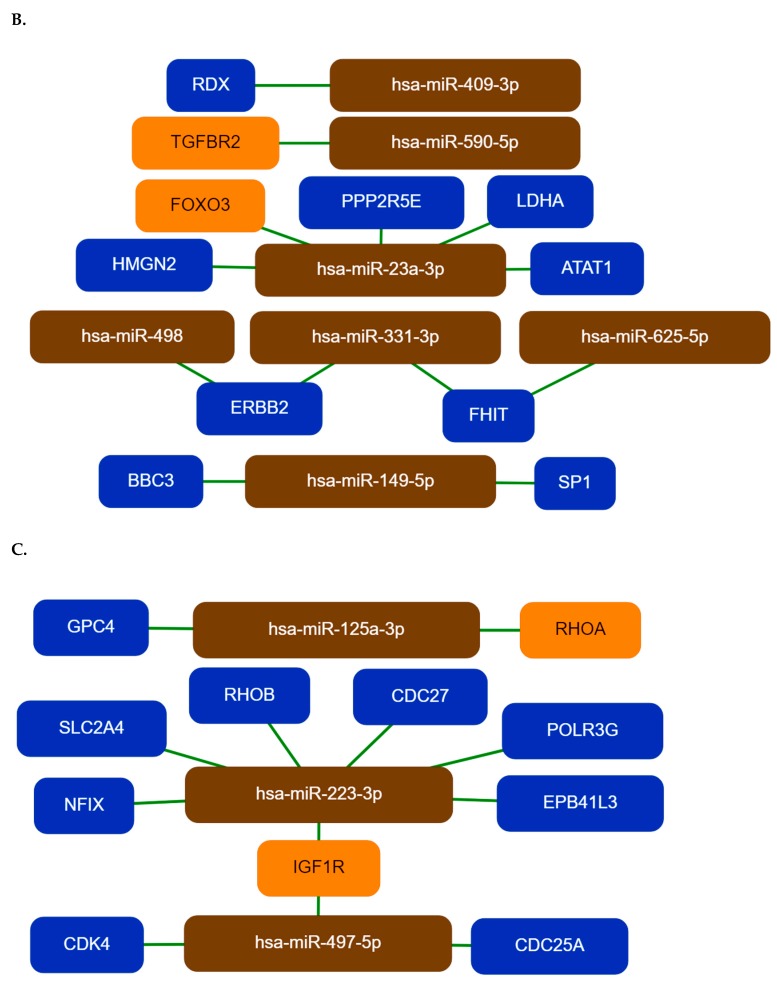
The core networks of differentially expressed miRNAs and their experimentally validated target genes. Group 1, Group 2, and Group 3 miRNAs and their interacting targets are represented in part **A**, **B**, and **C**, respectively. The networks were generated by the mirTargeLink tool using the strong interaction option. Isolated networks are also shown in the figure. Color code: Orange, more than two interactions; blue, two interactions in the full network.

To demonstrate and visualize the most important miRNA–target interactions, we have constructed core miRNA–target networks to show the strongest interactions among the differentially expressed miRNAs and target genes by the mirTargeLink tool (Figure 3). Just like in the miRNet network, hsa-miR-19b-3p, hsa-miR-26b-5p, hsa-miR-25-3p, and hsa-miR-301a-3p have central importance in the core network of Group 1 miRNAs. Their targets, PTEN, EZH2, KAT2B, BCL2L11, TP53, SMAD4, and ERBB2 are known to be involved in tumorogenesis (Figure 3A). 

Among Group 2 miRNAs, hsa-miR-23a-3p, hsa-miR-498, hsa-miR-331-3p, and hsa-miR-625-5p have central importance. Hsa-miR-23a-3p targets HMGN2, FOXO3, PPP2R5E, LDHA, and ATAT1. Hsa-miR-498, hsa-miR-331-3p, and hsa-miR-625-5p have two common targets, the tumor suppressor FHIT gene and the proto-oncogene ERBB2 (Figure 3B). In the third miRNA group (Group 3) hsa-miR-223-3p and hsa-miR-497-5p have a central role. Their common target IGF1R is a proto-oncogene, which is highly expressed in several tumor cells, CDK4 and CDC25A are proto-oncogenes (Figure 3C) [27,28,29,30]. 

**Figure 4:**
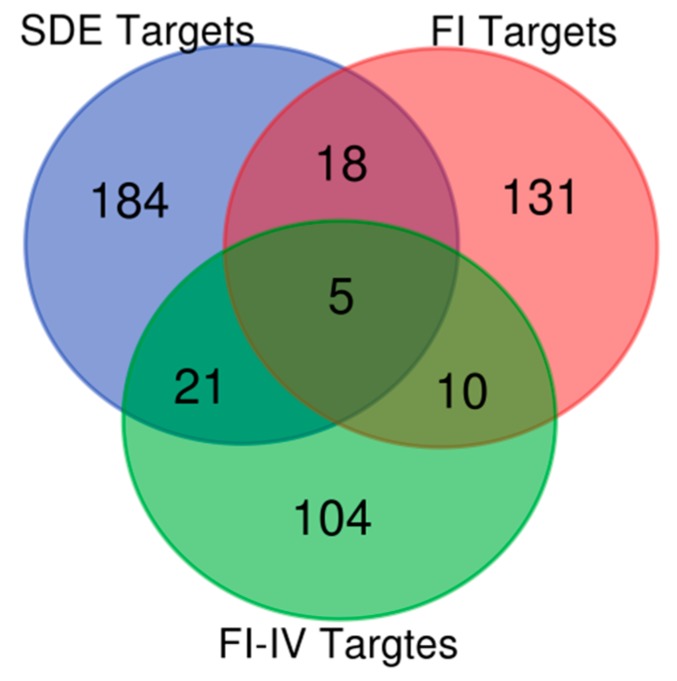
Venn diagram showing the common targets of the three differentially expressed miRNA groups. The SDE, FI, and FI-IV labels represent Group 1, Group 2, and Group 3 miRNAs, respectively. SDE: significantly differentially expressed miRNAs; FI: miRNAs expressed in FIGO I stage patients; FI-FIV: miRNAs expressed in all patients.

In addition to using miRNet, the TargetScan and miRTarBase databases were also used to predict target genes of the differentially expressed miRNAs, only experimentally validated targets were chosen from all three databases (Supplementary Table S2). In order to assess miRNA expression patterns in OC, we further analyzed our data to recognize common patterns of targets among the different samples to focus our analysis on their similarities. Intersections of common targets of the three differently expressed miRNA groups revealed 54 miRNA targets that were simultaneously differentially targeted by the different miRNA groups. Of these targets, we identified five genes that were targeted by all three miRNA groups. Figure 4 and Table 3 show the intersections of common targets of the three differently expressed miRNA groups. 

In Table 3, the HOTAIR lncRNA is also present as the target of miRNAs. This is not the only identified lncRNA target, significantly differentially expressed miRNAs interact with XIST, HOTAIR, MALAT1, NEFL, KCNQ1OT1, and CTA-204B4.6, as major lncRNA hubs in the network (data not shown). 

### 2.4. Pathway and Gene Ontology Enrichment Analysis of miRNA Targets

To obtain a more precise understanding of the potential pathophysiological role of the differentially expressed miRNAs in the OC development, a functional annotation and enrichment analysis of their target genes in the gene ontology biological process (GO-BP) terms and in canonical pathways (in the KEGG database) was performed using the database for annotation, visualization, and integrated discovery tool (DAVID) [31]. Our analysis resulted in a large number of enriched functional categories (pathways and GO terms, too), some of them very general including a large number of target genes such as hsa05200:Pathways in cancer, hsa05206:MicroRNAs in cancer, hsa04110:Cell cycle; or for the GO-BP terms: GO:0000165~MAPK cascade; GO:0045944~positive regulation of transcription from RNA polymerase II promoter; GO:0043066~negative regulation of apoptotic process or GO:0008283~cell proliferation. We have ranked the categories according to the significance of target enrichment and the top 25 KEGG pathways and GO-BP terms are shown in Supplementary Table S3. Several cancer types (glioma, prostate cancer, chronic myeloid leukemia, bladder cancer, non-small cell lung cancer, breast cancer, colorectal cancer, renal cell carcinoma, among others) and viral infectious pathways (Hepatitis C, HTLV-I infection, Epstein-Barr virus infection) were also identified in our analysis, however these categories are not included in the Supplementary table. Some of the enriched KEGG pathways and GO terms are shared by targets of more than one miRNA groups and these are shown in Table 4 and Table 5, respectively.

At the same time targets of the three differentially expressed miRNA groups are also enriched in pathways unique to a given miRNA group. These unique pathways are shown in Table 6.

### 2.5. Protein–Protein Interaction Network Analysis of miRNA Targets

**Figure 5:**
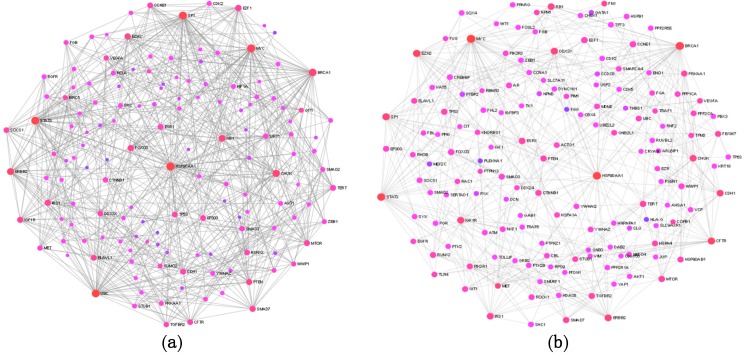
Topology of the general and ovary-specific protein–protein interaction (PPI) networks constructed from the common targets of differentially expressed miRNAs using the NetworkAnalyst tool. Part (**a**) and (**b**): The general and ovary-specific minimum PPI networks, respectively. Nodes represent proteins, only the major hub nodes are labeled in the networks.

Subsequently targets of the three different miRNA groups were collapsed into a single non-redundant target list, which was used to construct protein–protein interaction (PPI) networks. First, a general PPI network, then an ovary-specific PPI network was constructed from the target lists by using the NetworkAnalyst tool. Both networks proved to be a large fuzzy network, in the general PPI there are 3168 nodes and 5379 edges, the ovary-specific network contains 2353 edges and 3361 nodes. In Figure 5a,b, the general and ovary-specific minimum networks are shown with major hubs labeled, respectively.

The size of the nodes in Figure 5 corresponds to their degree centrality (and betweenness centrality) values and the nodes in the network with large degree centrality are considered to be key nodes or hubs with biological importance. Most of the major hubs overlap in the two networks and represent proteins, which are already known to be involved in tumorogenesis, such as HSP90AA1, MYC, SP1, BRCA1, RB1, CFTR, STAT3, E2F1, ERBB2, EZH2, and MET among others. The ranking of nodes based on degree centrality, however, is not identical in the two PPI networks: MYC, BRCA1, CFTR, EZH2, and STAT3 have higher ranks in the ovary-specific network. These interacting proteins occupy a central position in the network, so they could be considered as key biological factors in OC development. An advantage of the network-based approach is that it provides a mean to discover novel proteins, which interact physically and functionally with the seed proteins and may represent new cancer genes or cancer biomarkers.

The NetworkAnalyst tool could carry out a network-based functional enrichment and pathway analysis based on the gene ontology and KEGG databases, too. That way it was possible to compare results of the general analysis with results of an ovary-specific enrichment analysis. The results are shown in Table 7 and Table 8. 

These functional enrichment results suggest that circulating plasma miRNAs are not randomly released from cells since many of their predicted target genes are enriched in critically important pathways and biological processes contributing to tumorogenesis, such as TGFβ signaling pathway, NF-kappaB signaling pathway, VEGF signaling pathway, Rap1 signaling pathway, Ras signaling pathway, ErbB signaling pathway, Focal adhesion, MAPK signaling pathway FoxO signaling pathway, Proteoglycans in cancer, PI3K-Akt signaling pathway, Focal adhesion, AGE-RAGE signaling pathway, JNK cascade, Peptidyl-Tyr-phosphorylation, Phosphatidylinositol 3-kinase signaling, and SMAD protein signal transduction. In the case of OC, the estrogen signaling pathway could have specific importance [32]. The functional enrichment analysis also revealed several cancer types; however, those are not listed in Table 7 and Table 8.

## 3. Discussion

In recent years several groups examined the biological importance of cell free miRNAs present in body fluids. It was suggested that circulating miRNAs have the potential to become non-invasive biomarkers for the early diagnosis of cancer [9,10,24]. It is equally interesting however, to study the pathophysiological role of these miRNAs, since circulating miRNAs released from cells are known to be involved in intercellular communication and dysregulation of miRNAs in tissues is known to be associated with several cancers [7,9,10,14]. 

We have compared the expression profiles of circulating miRNA in blood plasma samples of six healthy females and 18 OC patients. Patients were divided into FIGO I, FIGO III, and FIGO IV stages, having six patients in each group. The nCounter Human v3 miRNA Panel of the NanoString System was used to measure the miRNA levels. MiRNA counts were low for most of the miRNAs, especially in the control samples. This might be due to the detection method, the NanoString method does not require an amplification step, so it is clearly different from those methods that use PCR for the miRNA measurement. It might suggest that the NanoString method is less sensitive for low abundance miRNAs.

Comparing miRNA expression profiles in the control and OC patient samples we have identified 45 miRNAs showing different expression between controls and patients. Our data showed that 17 miRNAs out of 45 were present both in the control and patient plasma (Group 1 miRNAs), however their expression levels differed significantly between the four groups. With the exception of the tumor suppressor has-miR-584-5p all Group 1 miRNAs were upregulated. 19 miRNAs were found only in samples of FIGO I patients (Group 2 miRNA) and nine miRNAs were detected in all three patient groups but were absent in control samples (Group 3 miRNAs). The finding of miRNAs, which are present only in patient samples might be important from a diagnostic point of view, as it shows that circulating miRNAs have the potential to be used as non-invasive biomarkers. Our sample number, however, is too low to draw any firm conclusions.

The large majority of our differentially expressed miRNAs have been previously reported to play a role in different cancer types. A few of our differentially expressed miRNAs, however, were found to be associated with OC in previous reports (i.e., hsa-miR-144-3p, hsa-miR-337-5p, hsa-miR-500a-5p, hsa-miR-26b-5p, hsa-miR-125a-3p, hsa-miR-19b-3p) [7,14,24,26]. Using the miRNet tool and a network-based approach we have constructed a miRNA–target interaction network for the miRNA groups. MiRNAs were ranked based on their degree-centrality value in the network, which reflects their biological importance. Hsa-mir-26b-5p, hsa-mir-19b-3p, and hsa-mir-25-3p were the top three ranked miRNAs for Group 1, hsa-mir-331-3p, hsa-mir-520g-3p, and hsa-mir-149-5p for Group 2 and hsa-mir-497-5p, hsa-mir-125a-3p, and hsa-mir-223-3p were the top three miRNAs for Group 3. The key miRNA–target interactions were visualized by the miTargetLink tool. For the Group 1 miRNAs, PTEN, EZH2, KAT2B, BCL2L11, TP53, SMAD4, and ERBB2 are the main targets, all known to be involved in tumorogenesis. PTEN and TP53 are known tumor suppressor proteins—impairing of KAT2B activity may contribute to genome instability; both oncogenic and tumor suppressive effects of EZH2 have been demonstrated in different cancer types and its expression is known to be regulated by miRNAs [33,34]. BCL2L11 is a tumor suppressor, it is an important regulator of apoptosis; loss of the SMAD4 activity may disrupt DNA damage response and repair mechanisms and enhance genomic instability [35,36]. The downregulation of these genes is in agreement with tumor formation, however the receptor tyrosine kinase ERBB2 is a proto-oncogene, the role of its downregulation by has-miR-25-3p and hsa-miR-552-3p is not known [37].

HMGN2, FOXO3, PPP2R5E, LDHA, ATAT1, FHIT, and the ERBB2 genes are the main targets for top Group 2 miRNAs, while the IGF1R, CDK4, CDC25A, SLC2A4/GLUT4, RHOB, CDC27, and POLR3G are the major targets for miRNAs of the third group. HMGN2 is an anti-tumor effector molecule of CD8^+^T cells, FOXO3 is a core tumor suppressor in breast cancer; downregulation of PPP2R5E is a common event in acute myeloid leukemia [38,39,40]. However, the ATAT1 activity is required for microtubule organization, it is specifically upregulated in colon cancer tissue and LDHA has an aberrantly high expression in multiple cancers [41,42]. 

Overexpression of CDC25A is known to be associated with malignancy and poor prognosis in cancer patients [30]. Hsa-miR-223-3p targets the EPB41L3, a potential tumor suppressor gene, the NFIX gene that regulates both cell proliferation and migration, the SLC2A4/GLUT4 is a glucose transporter and a biomarker for many types of malignant tumors [43,44,45]. A dualistic role of RHOB was reported, it could be a proto-oncogene or a tumor suppressor depending on the context of cancer development and progression. CDC27 is a tumor suppressor, its downregulation inhibits the proliferation of cancer cells [46], POLR3G is required for proliferation, its depletion triggers proliferative arrest and differentiation of prostate cancer cells [47]. 

Considering the negative regulatory role of miRNAs, it is noteworthy to find that not only tumor suppressor genes but proto-oncogenes are also present among the interacting targets. However, it is known, that miRNAs could have dualistic effect, a given miRNA can be an oncomiR or tumor suppressor depending on the cellular context [23]. The miRTarBase and TargetLink databases were also used to predict experimentally validated targets of the differentially expressed miRNAs, several common targets were identified for three miRNA groups, the MET, SMAD7, EZH2, TERT, and IL6 genes for example are targeted by at least one member of each different miRNA group. The tyrosine–protein kinase MET is a proto-oncogene, its role in cell migration and in epithelial–mesenchymal transition (EMT) is well known [48]. SMAD7 has a tumor suppressing role through blocking the TGF-β-stimulated cancer progression by increasing angiogenesis and inducing EMT [49]. The telomerase reverse transcriptase TERT gene plays a crucial step in tumorogenesis, it is required to maintain the telomere length and telomerase activity to gain immortality [50]. IL6 is an inflammatory cytokine which promotes metastasis in OC [51].

The miRNA group specific target lists were used in a functional annotation analysis based on the enrichment of miRNA targets in the KEGG pathways and gene ontology biological processes terms. This analysis revealed that miRNA targets are enriched in known cancer pathways, signaling pathway which are crucial pathways in tumorogenesis. To name a few, FoxO signaling pathway, p53 signaling pathway, PI3-AKT pathway AMPK pathway, TGFβ signaling pathway, focal adhesion, proteoglycans in cancer, Hippo signaling pathway, ERBB signaling pathway, JAK-STAT signaling pathway, Estrogen signaling pathway, and MAPK signaling pathway were among the most significant ones. In GO-BP terms the positive regulation of gene expression, positive regulation of cell proliferation, negative regulation of cell proliferation, cellular response to hypoxia, negative regulation of transcription, positive regulation of tyrosine phosphorylation of Stat3 protein, and negative regulation of apoptotic process were the most significant ones based on target enrichment and over-representation. All of these processes and terms are known players of tumorogenesis. The results of the enrichment analysis show that most miRNA targets are involved in signaling pathways and biological processes, which are critical for tumor formation, suggesting that circulating miRNAs could be potential regulatory factors in tumorogenesis. At the same time these data also show that the identified enriched pathways and GO terms are not specific for a given tumor type, our identified miRNA targets are associated with regulatory and signaling processes which are important in several different tumor types.

The target lists generated by the three prediction tools were merged into a single list and possible interactions between the target proteins and their functionally important interacting protein partners were analyzed by constructing general and ovary-specific PPI networks. The major hub proteins—HSP90AA1, MYC, SP1, BRCA1, RB1, CFTR, STAT3, E2F1, ERBB2, EZH2, and MET—were basically the same in the two networks, suggesting that our differentially expressed miRNAs regulate target genes which are involved in basic processes of tumor formation. This notion is strengthened by our network-based functional enrichment analysis, which provided a very similar enrichment pattern (both in KEGG and GO-BP terms) to those which were recognized by the DAVID tool for the different miRNA target groups. 

In conclusion, our pilot study identified significantly differentially expressed circulating miRNAs in plasma samples of OC patients. Our functional annotation analysis showed that the experimentally validated targets of the differentially expressed miRNAs are key regulators of tumor formation, suggesting that circulating miRNAs might play an important pathophysiological role in the formation of different tumor types. On the other hand, these results also show that the differentially expressed miRNAs identified in our study have limited usefulness in the diagnosis of OC. A clear limitation of our study is the low sample size, however, we feel that our results would warrant validation in a large cohort of OC patients.

## 4. Materials and Methods

### 4.1. Patients and Samples

Twenty-four blood samples (six disease-free healthy controls, 18 serous ovarian cancer patients) were collected from the Department of Obstetrics and Gynecology, Faculty of Medicine, University of Debrecen. All patients that underwent surgery and tissue samples were histologically diagnosed. Pathological characterization of tumor stages was assessed according to the International Federation of Gynecology and Obstetrics (FIGO) criteria. None of the patients received chemotherapy or radiotherapy treatment prior to participation in the study. Each subject provided written informed consent. The study was approved by the Scientific and Research Ethics Committee of the Medical Research Council of the Ministry of Health, Budapest, Hungary (ETT TUKEB), (Project Identification Code: 30231-2/2016/EKU, date: 06 June 2016) and was conducted in accordance with the Declaration of Helsinki. Controls were followed up, none of them received gynecological treatment during the study period.

Peripheral blood (9 mL) was drawn into EDTA anticoagulated tubes (BD Vacutainer) from each patient and from healthy volunteers and kept at 4 °C until further processing (within two hours of collection). Plasma samples were subjected to a two-step centrifugation protocol (2500× *g* and 16,000× *g*; 10–10 min, 4 °C) to obtain plasma. After separation, the cell-free plasma samples were homogenized, aliquoted, and stored at −80 °C until further processing.

### 4.2. RNA Isolation and Purification for the NanoString Device

Prior to RNA isolation blood samples were thawed on ice, then circulating RNA was isolated from 500 µL plasma samples using the miRNeasy Serum/Plasma RNA isolation kit (Qiagen, Hilden, Germany) according to the manufacturer’s protocol. The quality of the RNA was analyzed using the Nanodrop device (Thermo Scientific, Waltham, MA, USA).

### 4.3. RNA Expression Analysis

The miRNA content of all samples was analyzed using the nCounter Human v3 miRNA Panel of NanoString nCounter Analysis System (NanoString Technologies, Seattle, WA, USA), which contains 798 unique hsa-miRNA barcodes. 100 ng RNA/sample was used as input for the measurements, hybridization was carried out for 18 h, and miRNA counts were collected by scanning on the HIGH mode. The background correction of data was performed by subtracting the mean ± 2 standard deviation of the negative control set. Lane-by-lane technical variation was corrected by using the geometric median value of the positive code-set. The complete data set was normalized by calculating the geometric mean of 10 “housekeeping” miRNA counts for each sample to generate the normalization factor. 

### 4.4. Prediction and Analysis of Experimentally Validated Target Genes

First, a miRNA–target gene network was constructed using the web based miRNet tool [http://www.mirnet.ca]. Top miRNAs in the network were ranked by degree and betweenness centrality values. The prediction of experimentally validated target genes of miRNAs was carried out by using the web based miRNet, miRTarBase, and TargetScan software programs (http://miRTarBase.mbc.nctu.edu; www.targetscan.org). Target intersections were further validated by the miRWalk2 database (www. http://zmf.umm.uni-heidelberg.deg). The general and ovary-specific protein–protein interaction (PPI) network of target genes was constructed using the NetworkAnalyst 3.0 tool [www.networkanalyst.ca]. 

### 4.5. Functional Annotation and Pathway Enrichment Analysis

The lists of miRNA targets was used as input and the online Database for Annotation, Visualization, and Integrated Discovery (DAVID; https://david.ncifcerf.gov) software tool was used to perform gene ontology (GO) and Kyoto Encyclopedia of Genes and Genomes (KEGG) based functional pathway enrichment analysis for the predicted target genes of prioritized differentially expressed hsa-miRNAs. The NetworkAnalyst tool was used to carry out ovary-specific enrichment analysis. A p-value of < 0.05 was considered statistically significant. 

### 4.6. Validation of hsa-miRNA Expression by Quantitative Real-Time Polymerase Chain Reaction (RT-qPCR) on Selected hsa-miRNAs

Circulating RNA was extracted from 200 µL plasma samples of 16 healthy control females and 18 OC patients by using the miRNeasy Serum/Plasma Kit (Qiagen, Hilden, Germany) including 3.5 µL miRNeasy Serum/Plasma Spike-In Control RNA, according to the manufacturer’s instructions. A miRNA-specific fluorometric assay on a Qubit^®^ 2.0 Fluorimeter (Thermo Fischer Scientific, USA) was used to determine the concentration of RNA. To detect and measure the amounts of mature miRNAs the miScript PCR System (Qiagen, Hilden, Germany) was used. The miScript II RT Kit (Qiagen) was used for reverse transcription of miRNAs. The quantitative real-time PCR reaction was used (LightCycler^®^96; Roche Molecular Systems Inc., Pleasanton, CA, USA) to determine the level of hsa-miR-25-3p, hsa-miR-26b-5p, hsa-miR-144-3p, hsa-miR-19b-3p, hsa-miR-301a-3p, hsa-miR-148b-3p, hsa-miR-553, and hsa-miR-197 by using the miScript SYBR Green PCR Kit (Qiagen). The PCR reaction mixture contained 500 pg reverse transcription products. The reaction mixtures were first denatured at 95 °C for 15 min, followed by 50 amplification cycles of 94 °C for 15 s, 55 °C for 30 s and 70 °C for 30 s. Finally, a melting curve was generated by taking fluorescent measurements every 0.2 °C for 25 s from 50 °C until 95 °C to detect a single PCR product. Cycle threshold (Ct) values above the determinable range (up to 45) were assigned a Ct of 45. All measurements were performed in triplicate and the amounts of PCR products were normalized to an internal control (hsa-miR103-3p). Relative expression levels were calculated by the 2^−∆Ct^ method. 

### 4.7. Statistical Analysis

All data were analyzed using the GraphPad Prism statistical package (GraphPad Prism7, San Diego, CA, USA). Descriptive column statistics of each data set were performed and the distribution of data was analyzed by the Kolmogorov–Smirnov test. To assess the statistical significance of differences in miRNA counts between the control and patient groups the nonparametric one-way ANOVA Kruskal–Wallis test in combination with the post hoc Dunn’s test to adjust for multiple comparisons was applied. In all tests the difference was considered significant at *p <* 0.05 value. Where applicable, the Dunn’s p-values were indicated as: *p <* 0.05(*); *p <* 0.01(**). The fold change in the expression of a miRNA between the control data and a given FIGO stage data was calculated as: (FIGO stage mean count – Control mean count)/Control mean count. 

## Figures and Tables

**Table 1:** List of microRNAs (miRNAs) showing significantly different expression patterns among controls and the three ovarian cancer (OC) patient groups.

Group 1 ^1^	Group 2	Group 3
hsa-miRNA ID	hsa-miRNA ID	hsa-miRNA ID
hsa-miR-1185-2-3p	hsa-miR-1185-1-3p	hsa-miR-125a-3p
hsa-miR-553	hsa-miR-1197	hsa-miR-1281
hsa-miR-144-3p	hsa-miR-1266-5p	hsa-miR-128-2-5p
hsa-miR-146b-5p	hsa-miR-149-5p	hsa-miR-1305
hsa-miR-148b-3p	hsa-miR-23a-3p	hsa-miR-223-3p
hsa-miR-1976	hsa-miR-3161	hsa-miR-325
hsa-miR-19b-3p	hsa-miR-331-3p	hsa-miR-497-5p
hsa-miR-526a	hsa-miR-331-5p	hsa-miR-500a-5p
hsa-miR-219a-2-3p	hsa-miR-337-5p	hsa-miR-548h-3p
hsa-miR-25-3p	hsa-miR-3615	
hsa-miR-26b-5p	hsa-miR-409-3p	
hsa-miR-301a-3p	hsa-miR-4455	
hsa-miR-513a-3p	hsa-miR-498	
hsa-miR-552-3p	hsa-miR-520g-3p	
hsa-miR-584-5p	hsa-miR-584-5p	
hsa-miR-613	hsa-miR-590-5p	
hsa-miR-615-5p	hsa-miR-625-5p	
	hsa-miR-628-5p hsa-miR-651-5p	

^1^ Group 1: Significantly differentially expressed miRNAs. Group 2: MicroRNAs expressed only in FIGO I stage patients. Group 3: MicroRNAs expressed only in patients, but in all stages.

**Table 2:** Ranking of the differentially expressed miRNAs based on their degree centrality values in the miRNet network.

Group 1		Group 2		Group 3	
miRNA ID	Degree	miRNA ID	Degree	miRNA ID	Degree
hsa-mir-26b-5p	1874	hsa-mir-331-3p	406	hsa-mir-497-5p	461
hsa-mir-19b-3p	714	hsa-mir-520g-3p	404	hsa-mir-125a-3p	310
hsa-mir-25-3p	518	hsa-mir-149-5p	397	hsa-mir-548h-3p	292
hsa-mir-1976	501	hsa-mir-498	320	hsa-mir-1305	195
hsa-mir-148b-3p	403	hsa-mir-23a-3p	249	hsa-mir-1281	180
hsa-mir-301a-3p	395	hsa-mir-625-5p	227	hsa-mir-500a-5p	145
hsa-mir-144-3p	211	hsa-mir-4455	165	hsa-mir-223-3p	98
hsa-mir-513a-3p	187	hsa-mir-1185-1-3p	117	hsa-mir-325	32
hsa-mir-552-3p	167	hsa-mir-3161	115		6
hsa-mir-146b-5p	121	hsa-mir-409-3p	111		
hsa-mir-615-5p	70	hsa-mir-1197	74		
hsa-mir-584-5p	67	hsa-mir-584-5p	67		
hsa-mir-219a-2-3p	63	hsa-mir-590-5p	66		
hsa-mir-526a	61	hsa-mir-651-5p	65		
		hsa-mir-331-5p	63		
		hsa-mir-628-5p	51		
		hsa-mir-3615	39		
		hsa-mir-337-5p	7		

**Table 3:** Shared targets of the three differentially expressed miRNA groups.

Compared Groups	Common Targets	Genes
Gr1/Gr2/Gr3	5	MET SMAD7 EZH2 TERT IL6
Gr2/Gr1	18	TLR4 MTOR IGF1 ZEB1 SOX4 PTBP2 BIRC5 CCNE1 RHOB MMP16 IGF1R MYC PXK SOCS1 PBX3 PRKAA1 CFTR FBXW7
Gr1/Gr3	21	ROCK1 KCNJ6 PTEN TGFBR2 HOTAIR PLEKHA1 ERBB2 FGA CDH1 PPP2R5E FGG IRS1 RECK DDX3X FGB HLA-G WWP1 RB1 TCEAL1 HSP90AA1 ZFX
Gr2/Gr3	10	GIT1 NPM3 BRCA1 CHUK STAT3 SP1 FOXO3 VEGFA E2F1 MEF2C

**Table 4:** Functional annotation of target genes based on their enrichment in specific Kyoto Encyclopedia of Genes and Genomes (KEGG) pathways. The top 25 most significant pathways are shown, which are targeted by at least two miRNA groups.

Targets of All miRNA groups	Targets of Group 1 and Group 2 miRNAs	Targets of Group 2 and Group 3 miRNAs	Targets of Group 1 and Group 3 miRNAs
hsa04068:FoxO signaling pathway	hsa05213:Endometrial cancer	hsa04066:HIF-1 signaling pathway	hsa04110:Cell cycle
hsa04115:p53 signaling pathway	hsa04390:Hippo signaling pathway	hsa05206:MicroRNAs in cancer	hsa04012:ErbB signaling pathway
hsa04151:PI3K-Akt signaling pathway	hsa04722:Neurotrophin signaling pathway	hsa04621:NOD-like receptor signaling pathway	hsa04150:mTOR signaling pathway
hsa04152:AMPK signaling pathway		hsa04550:Signaling pathways regulating pluripotency of stem cells	hsa04917:Prolactin signaling pathway
hsa04350:TGF-beta signaling pathway		hsa05202:Transcriptional misregulation in cancer	
hsa04510:Focal adhesion			
hsa04931:Insulin resistance			
hsa05200:Pathways in cancer			
hsa05205:Proteoglycans in cancer			
hsa05230:Central carbon metabolism in cancer			

**Table 5:** Functional annotation of target genes based on their enrichment in gene ontology (GO_ biological processes. Those members of the top 25 GO terms are shown, which are enriched by all three miRNA group targets.

GO Biological Process
GO:0010628~positive regulation of gene expression
GO:0008284~positive regulation of cell proliferation
GO:0008285~negative regulation of cell proliferation
GO:0071456~cellular response to hypoxia
GO:0045892~negative regulation of transcription, DNA-templated
GO:0042517~positive regulation of tyrosine phosphorylation of Stat3 protein
GO:0043066~negative regulation of apoptotic process

**Table 6:** The unique functional annotation of target genes of a given miRNA group based on their enrichment in specific KEGG pathways.

Targets of Group 1 miRNAs	Targets of Group 2 miRNAs	Targets of Group 3 miRNAs
hsa04919:Thyroid hormone signaling pathway	hsa04920:Adipocytokine signaling pathway	hsa04630:Jak-STAT signaling pathway
hsa05203:Viral carcinogenesis	hsa04520:Adherens junction	hsa04910:Insulin signaling pathway
hsa04620:Toll-like receptor signaling pathway	hsa04210:Apoptosis	hsa04660:T cell receptor signaling pathway
hsa04014:Ras signaling pathway	hsa04922:Glucagon signaling pathway	hsa04062:Chemokine signaling pathway
hsa04015:Rap1 signaling pathway	hsa04064:NF-kappa B signaling pathway	hsa04914:Progesterone-mediated oocyte maturation
hsa04071:Sphingolipid signaling pathway	hsa04915:Estrogen signaling pathway	
	hsa04010:MAPK signaling pathway	

**Table 7:** KEGG pathways-based general and ovary-specific functional enrichment analysis of all target genes of differentially expressed miRNAs.

General Analysis ^1^		Ovary-Specific Analysis	
KEGG Pathway	P Value	KEGG Pathway	P Value
Pathways in cancer	2.3564 × 10^−29^	Pathways in cancer	2.26 × 10^−38^
Central carbon metabolism in cancer	3.8587 × 10^−11^	Epstein-Barr virus infection	1.54 × 10^−29^
Endometrial cancer	1.1410 × 10^−8^	Cell cycle	5.79 × 10^−25^
Insulin resistance	5.5166 × 10^−8^	Cellular senescence	1.3 × 10^−24^
TGF-beta signaling pathway	2.0410 × 10^−7^	ErbB signaling pathway	3.01 × 10^−24^
Toll-like receptor signaling path.	2.1829 × 10^−7^	MAPK signaling pathway	5.21 × 10^−24^
NF-kappa B signaling pathway	3.3876 × 10^−7^	FoxO signaling pathway	3.67 × 10^−23^
AMPK signaling pathway	4.6513 × 10^−7^	Proteoglycans in cancer	8.67 × 10^−23^
Prolactin signaling pathway	7.7049 × 10^−7^	Ubiquitin mediated proteolysis	1.7 × 10^−21^
Ras signaling pathway	1.4145 × 10^−6^	PI3K-Akt signaling pathway	2.24 × 10^−20^
ErbB signaling pathway	1.8772 × 10^−6^	Prolactin signaling pathway	3.53 × 10^−20^
Focal adhesion	2.9277 × 10^−6^	Focal adhesion	7.12 × 10^−20^
mTOR signaling pathway	2.8262 × 10^−6^	AGE-RAGE signaling pathway	4.55 × 10^−18^
Insulin signaling pathway	1.1171 × 10^−5^	T cell receptor signaling pathway	1.02 × 10^−16^
T cell receptor signaling pathway	1.1184 × 10^−5^	Adherens junction	1.24 × 10^−16^
Chemokine signaling pathway	6.4640 × 10^−5^	TNF signaling pathway	1.47 × 10^−16^
VEGF signaling pathway	9.9975 × 10^−5^	Estrogen signaling pathway	2.35 × 10^−16^
cAMP signaling pathway	1.2228 × 10^−4^	NF-kappa B signaling pathway	3.44 × 10^−16^
Rap1 signaling pathway	1.7607 × 10^−4^	Transcriptional regulation in cancer	3.8 × 10^−16^
Estrogen signaling pathway	0.0022	TGF-beta signaling pathway	1.84 × 10^−14^

^1^ In general analysis tissue specific expression is not considered.

**Table 8:** Gene ontology-based general and ovary-specific functional enrichment of all target genes of differentially expressed miRNAs.

General Analysis ^1^		Ovary-Specific Analysis	
GO Biological Process	P Value	GO Biological Process	P Value
Phosphatidylinositol-mediated signaling	4.5190 × 10^−11^	Phosphorylation	4.62 × 10^−61^
TGFβ receptor signaling pathway	1.9554 × 10^−8^	Regulation of protein modification process	1.14 × 10^−51^
MAPK cascade	4.6884 × 10^−7^	Regulation of transferase activity	2.69 × 10^−46^
Peptidyl-Tyr-phosphorylation	6.050 × 10^−7^	Regulation of kinase activity	2.5 × 10^−45^
Phosphatidylinositol 3-kinase signaling	7.1633× 10^−7^	Enzyme linked receptor protein signaling	1.45 × 10^−43^
Positive regulation of Tyr- phosphorylation of Stat3 protein.	9.8981 × 10^−7^	Regulation of cell cycle	1.46 × 10^−41^
I-κB kinase/NF-kappaB signaling	2.9099 × 10^−6^	Regulation of protein kinase activity	6.19 × 10^−41^
IL6-mediated signaling pathway	5.8361 × 10^−6^	Cell proliferation	2.65 × 10^−40^
Positive regulation of pri-miRNA transcription	1.2812 × 10^−7^	Cellular response to stress	1.15 × 10^−37^
Positive regulation of EMT	2.7940 × 10^−5^	Intracellular protein kinase cascade	2.57 × 10^−37^
JNK cascade	5.6629 × 10^−5^	Cell cycle	3.26 × 10^−37^
Response to calcium ion	1.4710 × 10^−4^	Positive regulation of RNA metabolic process	1.38 × 10^−36^
SMAD protein signal transduction	2.0946 × 10^−4^	Regulation of programmed cell death	3.09 × 10^−35^
Insulin signaling pathway	2.0956 × 10^−4^	Positive regulation of signal transduction	4.45 × 10^−35^
T cell receptor signaling pathway	2.9929 × 10^−4^	Regulation of cell proliferation	1.18 × 10^−34^
Positive regulation of GTPase activity	8.5273 × 10^−4^	Negative regulation of apoptotic process	6.4 × 10^−34^
Toll-like receptor signaling pathway	9.5585 × 10^−4^	Intracellular signal transduction	8.73 × 10^−34^
Cell-matrix adhesion	0.0010	Reproduction	8.6 × 10^−33^
Heterotypic cell-cell adhesion	0.0012	Negative regulation of transcription	1.78 × 10^−32^
SMAD protein complex assembly	0.0017	Negative regulation of nucleobase_containing compound metabolic process	3.1 × 10^−29^

^1^ In general analysis tissue specific expression is not considered.

## Supplementary Materials

Supplementary materials can be found at https://www.mdpi.com/1422-0067/20/18/4533/s1.

## Abbreviations

ANOVA	Analysis of variance
EMT	Epithelial–mesenchymal transition
FIGO	Multidisciplinary Digital Publishing Institute
GO	Gene ontology
KEGG	Kyoto Encyclopedia of Genes and Genomes
OC	Ovary cancer
PPI	Protein–protein interaction
