# Supplementary material for: Circulating miRNA Profiling in Plasma Samples of Ovarian Cancer Patients
Source: Int J Mol Sci. 2019 Sep 13;20(18):4533. doi: 10.3390/ijms20184533 (PMC6769773; doi:10.3390/ijms20184533)
Supplement: Supplementary file 1 [file ijms-20-04533-s001.zip › Supplementary Table 1.pdf]

Supplementary Table I.

Table 1. Demographic and clinicopathological data of the patients.

| Stage | Name | | Age | Tumor | Histology |
|---|---|---|---|---|---|
| Controls | C54 | | 62 | - | - |
| | C63 | | 60 | - | - |
| | C69 | | 56 | - | - |
| | C71 | | 54 | - | - |
| | C84 | | 53 | - | - |
| | C53 | | 70 | - | - |
| | | **Mean** | **59** | | |
| | | **SD** | **6,3** | | |
| | | | | | |
| FIGO IV | U50 | | 75 | Ovarium cc | Cystadenocarcinoma papillare serosum |
| | U52 | | 63 | Ovarium cc | Cystadenocarcinoma papillare serosum |
| | U69 | | 63 | Ovarium cc | Cystadenocarcinoma papillare serosum |
| | U46 | | 50 | Ovarium | Adenocarcinoma papillare serosum |
| | U66 | | 52 | Ovarium cc | Cystadenocarcinoma papillare serosum |
| | U27 | | 45 | Ovarium cc | Cystadenocarcinoma papillare serosum |
| | | **Mean** | **58** | | |
| | | **SD** | **11** | | |
| | | | | | |
| FIGO III | U41 | | 62 | Ovarium cc | Cystadenocarcinoma papillare serosum |
| | U49 | | 56 | Ovarium cc | Cystadenocarcinoma papillare serosum |
| | U55 | | 51 | Ovarium cc | Cystadenocarcinoma papillare serosum |
| | U56 | | 57 | Ovarium cc | Adenocarcinoma partim papillare serosum |
| | U67 | | 60 | Ovarium cc | Cystadenocarcinoma papillare serosum |
| | U25 | | 73 | Ovarium cc | Cystadenocarcinoma papillare serosum |
| | | **Mean** | **60** | | |
| | | **SD** | **7,5** | | |
| | | | | | |
| Figo I | U60 | | 61 | Ovarium cc | Cystadenocarcinoma papillare serosum |
| | U53 | | 62 | Ovarium cc | Cystadenocarcinoma papillare serosum |
| | U61 | | 72 | Ovarium cc | Cystadenocarcinoma papillare serosum |
| | U81 | | 43 | | Cystadenocarcinoma papillare serosum |
| | U86 | | 43 | | Cystadenocarcinoma papillare serosum |
| | U62 | | 28 | Ovarium cc | Cystadenocarcinoma papillare serosum |
| | | **Mean** | **52** | | |
| | | **SD** | **16** | | |
