# Supplementary material for: Circulating miRNA Profiling in Plasma Samples of Ovarian Cancer Patients
Source: Int J Mol Sci. 2019 Sep 13;20(18):4533. doi: 10.3390/ijms20184533 (PMC6769773; doi:10.3390/ijms20184533)
Supplement: Supplementary file 1 [file ijms-20-04533-s001.zip › Supplementary Table 2.pdf]

Table 2. Targets of differentially expressed miRNAs

Group 1 targtes	Group 2 targtes	Group 3 targtes	Combined_targets
ABCA1	AASDHPPT	ABCB1	AASDHPPT
ABCA2	ABRA	AGO1	ABCA1
ABCF2	APAF1	AKAP11	ABCA2
ACVR1	ATAT1	AMOT	ABCB1
ACSL4	ATF3	ANKEF1	ABCF2
Adamts1	BBC3	ANLN	ABRA
ALCAM	BRCA1	AP5Z1	ACVR1
ALKBH6	BTG2	ARTN	ACSL4
APP	C16orf72	ASCL1	Adamts1
ARHGEF28	C20orf24	ATM	AGO1
ARID4B	C5orf15	AURKB	AKAP11
ARL4C	CDH1	BCL2	ALCAM
ATP2A2	CELF1	BIRC5	ALKBH6
ATXN1	CHL1	BMP4	AMOT
BACE1	CHUK	BRCA1	ANKEF1
BCL2L11	CREB5	BTRC	ANLN
BCL3	CXCL12	CAPRIN1	AP5Z1
BIRC5	CXCL8	CARM1	APAF1
BLCAP	DAPK2	CCL3	APP
BMPR2	DDX3X	CCND3	ARHGEF28
BTBD3	DOHH	CCNE1	ARID4B
BTN2A2	E2F1	CDC25A	ARL4C
CASQ1	ERBB2	CDC27	ARTN
CASR	EZH2	CDC37L1	ASCL1
CCKBR	FANCG	CDK2	ATAT1
CCL26	FAS	CDK4	ATF3
CCNE1	FASLG	CFTR	ATM
CDC14A	FGA	CHEK1	ATP2A2
CDH1	FGB	CHUK	ATXN1
CDK6	FGF21	CLUAP1	AURKB
CDKN1A	FGFR1	COPS6	BACE1
CDKN1C	FGG	COPS8	BBC3
CEP350	FHIT	CTDNEP1	BCL2
CFTR	FOXA1	CXCL2	BCL2L11
CHORDC1	FOXM1	CYB5A	BCL3
COL1A2	FOXO3	CYLD	BIRC5
COX2	FZD5	DEPDC1	BLCAP
CPEB1	G6PC	DICER1	BMP4
CTGF	GABRG1	DPYSL5	BMPR2
CUL5	GIT1	E2F1	BRCA1
CYBB	GJA1	E2F3	BTBD3

CYP19A1	GLS	ECT2	BTG2
CYP2B6	GPC1	EFHD2	BTN2A2
CSF1	HES1	EIF4E	BTRC
DDX3X	HIP1R	EPB41L3	C16orf72
DHFR	HLA-G	ESRRA	C20orf24
DNMT1	HMGA1	EZH2	C5orf15
DSC2	HMGB2	FAM43A	CAPRIN1
DUSP5	HMGN2	FBXW7	CARM1
EGFR	HNF1B	FOXO1	CASQ1
EIF4A2	HOTAIR	FOXO3	CASR
EPHA2	HOXB4	FYN	CCKBR
ERBB2	HOXB7	GIT1	CCL26
ERBB4	HRG	GPC4	CCL3
ESR1	HSP90AA1	GPS1	CCND3
ESRRB	IGF2BP1	GXYLT2	CCNE1
ETS1	IL6	HAX1	CDC14A
EZH2	IL6R	HDGF	CDC25A
FAM218A	ILF3	HSP90B1	CDC27
FBXW7	ILK	HSPA4L	CDC37L1
FGA	ING5	IGF1	CDH1
FGB	IRF1	IGF1R	CDK2
FGF2	IRS1	IKBKB	CDK4
FGG	KCNJ6	IL13	CDK6
FH	KLF3	IL2	CDKN1A
FOXC1	KRTAP5-9	IL23R	CDKN1C
FUT11	LAMP1	IL6	Celf1
GATA4	LDHA	INHBA	CEP350
GCLC	LDHB	IRF4	CFTR
GCM1	LPAR1	ITPRIPL2	CHEK1
GSTP1	LRP5	KCNN4	CHL1
GRIK3	MEF2C	KHSRP	CHORDC1
GRK4	MET	LIF	CHUK
HAND2	MMP2	LMO2	CLUAP1
HAS2	MT2A	LRRC55	COL1A2
HGF	MTHFR	LRRC58	COPS6
HIPK1	MYD88	MACC1	COPS8
HIPK3	MYH1	MAFB	COX2
HLA-G	MYH2	MAFK	CPEB1
HNRNPD	MYH4	MAP2K1	CREB5
HOTAIR	NEK6	MEF2C	CTDNEP1
HSP90AA1	NPM3	MET	CTGF
IGF1	NRP2	MMP16	CUL5
IGF1R	NTRK3	MSMO1	CXCL12
IGF2	OLR1	MTA1	CXCL2

IL6	PABPC3	MTFR1L	CXCL8
IL6ST	PHLPP1	MTOR	CYB5A
IRAK1	PLEKHA1	MYC	CYBB
IRS1	POU4F2	MYL9	CYLD
ITGA5	PPARGC1A	NCKAP1	CYP19A1
JAG1	PPM1F	NEUROG1	CYP2B6
KAT2A	PPP1R1A	NFIA	CSF1
KAT2B	PPP2R2A	NFIX	DAPK2
KCNJ6	PPP2R5E	NHLRC3	DDX3X
KDELR1	PPP3R1	NLRP3	DEPDC1
KIT	PRDX3	NPM3	DHFR
KLF4	PRNP	NRG1	DICER1
KPNA2	PRPF8	NWD1	DNMT1
LARP1	PTEN	OTUD7B	DOHH
LATS2	PTGER2	PARP1	DPYSL5
LHCGR	PTPN11	PAX6	DSC2
LONRF3	RB1	PBX3	DUSP5
MALAT1	RBFOX2	PDE6A	E2F1
MAP2K4	RECK	PHACTR4	E2F3
MAP3K5	RECQL	PHLDA3	ECT2
MAP3K8	RGS5	PIGO	EFHD2
MAP3K9	ROCK1	PLEKHG3	EGFR
MDM2	Runx2	POLR3A	EIF4A2
MED28	SCAI	POLR3G	EIF4E
MEOX2	SEC62	PRDM1	EPB41L3
MET	SMAD3	PRKAA1	EPHA2
MID1IP1	SMAD5	PTBP2	ERBB2
MIEN1	SMAD7	PXK	ERBB4
MLH1	SOCS6	RAF1	ESR1
MMP16	SOX2	RBM23	ESRRA
MSANTD4	SP1	Reck	ESRRB
MTMR6	SPRY2	RHOA	ETS1
MTOR	SREK1	RHOB	EZH2
MTUS1	SRP54	RNF41	FAM218A
MXD1	ST7L	RPS6KB1	FAM43A
MYBL2	STAT3	RRAS2	FANCG
MYC	TCEAL1	RSL1D1	FAS
MYCN	TERF2	RUNX2	FASLG
MYLIP	TERT	SAR1A	FBXW7
NAMPT	TGFBR2	SCARB1	FGA
NCOA3	TMEM64	SDK1	FGB
NDFIP1	TMPO	SEMA3A	FGF2
NFE2L2	TOP1	SF3B1	FGF21
NFKB1	TRIM63	SIT1	FGFR1

NKRF	TSC1	SKI	FGG
NOTCH1	UNC13A	SLC25A51	FH
NOVA1	VEGFA	SLC2A4	FHIT
NR2C2	VMP1	SMAD7	FOXA1
NRAS	WWP1	SMU1	FOXC1
NUP54	XIAP	SMURF1	FOXM1
PAPD7	ZBTB2	SOCS1	FOXO1
PAX8	ZC3H4	SOX4	FOXO3
PBX3	ZFHX4	SP1	FUT11
PCSK2	ZFX	SP3	FYN
PDE4A	ZNF367	SSRP1	FZD5
PDGFRA		STAT1	G6PC
PIK3CA		STAT3	GABRG1
PIK3R2		STAT5A	GATA4
PIK3R3		STMN1	GCLC
PITX1		STRN3	GCM1
PIWIL4		SUGT1	GIT1
PKNOX1		TAL1	GJA1
PLAG1		TAX1BP1	GLS
PLEKHA1		TERT	GPC1
PLOD2		TGOLN2	GPC4
POP7		TLR4	GPS1
PPP2R5E		TNFRSF13C	GRIK3
PRKAA1		TOX	GRK4
PRKAB1		TRIM72	GSTP1
PRMT5		TTC4	GXYLT2
PSG4		TWIST1	HAND2
PTBP2		UBR3	HAS2
PTEN		USP48	HAX1
PTGS2		VEGFA	HDGF
PXK		WEE1	HES1
RAB24		WNT7A	HGF
RAN		XBP1	HIP1R
RARB		ZEB1	HIPK1
RB1		ZKSCAN1	HIPK3
RECK			HLA-G
REM1			HMGA1
REV3L			HMGB2
RGS3			HMGN2
RHOB			HNF1B
RNASE1			HNRNPD
ROCK1			HOTAIR
RPS27A			HOXB4
RUNX3			HOXB7

SEMA4C	HRG
SERPINE1	HSP90AA1
SGSM3	HSP90B1
SIRPB2	HSPA4L
SLC2A1	IGF1
SLC30A7	IGF1R
SLC5A5	IGF2
SMAD1	IGF2BP1
SMAD4	IKBKB
SMAD7	IL13
SNIP1	IL2
SOCS1	IL23R
SOX4	IL6
SPARC	IL6R
ST13	IL6ST
ST8SIA4	ILF3
SWT1	ILK
TAB1	ING5
TCEAL1	INHBA
TERT	IRAK1
TGFB1	IRF1
TGFBR2	IRF4
TIMP2	IRS1
TLR2	ITGA5
TLR4	ITPRIPL2
TMEM107	JAG1
TMF1	KAT2A
TNFAIP3	KAT2B
TNFSF10	KCNJ6
TP53	KCNN4
TPPP	KDELR1
TRAF5	KHSRP
TRAF6	KIT
TTC9C	KLF3
TTN	KLF4
TUG1	KPNA2
UHRF1	KRTAP5-9
ULK2	LAMP1
USP34	LARP1
USP9X	LATS2
UVRAG	LDHA
VAV3	LDHB
WDFY2	LHCGR
WDR4	LIF

WNK3	LMO2
WNT1	LONRF3
WWP1	LPAR1
XIST	LRP5
ZEB1	LRRC55
ZEB2	LRRC58
ZFX	MACC1
ZIC5	MAFB
ZNF154	MAFK
ZNF772	MALAT1
ZNRF3	MAP2K1
	MAP2K4
	MAP3K5
	MAP3K8
	MAP3K9
	MDM2
	MED28
	MEF2C
	MEOX2
	MET
	MID1IP1
	MIEN1
	MLH1
	MMP16
	MMP2
	MSANTD4
	MSMO1
	MT2A
	MTA1
	MTFR1L
	MTHFR
	MTMR6
	MTOR
	MTUS1
	MXD1
	MYBL2
	MYC
	MYCN
	MYD88
	MYH1
	MYH2
	MYH4
	MYL9
	MYLIP

NAMPT
NCKAP1
NCOA3
NDFIP1
NEK6
NEUROG1
NFE2L2
NFIA
NFIX
NFKB1
NHLRC3
NKRF
NLRP3
NOTCH1
NOVA1
NPM3
NR2C2
NRAS
NRG1
NRP2
NTRK3
NUP54
NWD1
OLR1
OTUD7B
PABPC3
PAPD7
PARP1
PAX6
PAX8
PBX3
PCSK2
PDE4A
PDE6A
PDGFRA
PHACTR4
PHLDA3
PHLPP1
PIGO
PIK3CA
PIK3R2
PIK3R3
PITX1
PIWIL4

PKNOX1
PLAG1
PLEKHA1
PLEKHG3
PLOD2
POLR3A
POLR3G
POP7
POU4F2
PPARGC1A
PPM1F
PPP1R1A
PPP2R2A
PPP2R5E
PPP3R1
PRDM1
PRDX3
PRKAA1
PRKAB1
PRMT5
PRNP
PRPF8
PSG4
PTBP2
PTEN
PTGER2
PTGS2
PTPN11
PXK
RAB24
RAF1
RAN
RARB
RB1
RBFOX2
RBM23
RECK
RECQL
REM1
REV3L
RGS3
RGS5
RHOA
RHOB

RNASE1
RNF41
ROCK1
RPS27A
RPS6KB1
RRAS2
RSL1D1
RUNX2
RUNX3
SAR1A
SCAI
SCARB1
SDK1
SEC62
SEMA3A
SEMA4C
SERPINE1
SF3B1
SGSM3
SIRPB2
SIT1
SKI
SLC25A51
SLC2A1
SLC2A4
SLC30A7
SLC5A5
SMAD1
SMAD3
SMAD4
SMAD5
SMAD7
SMU1
SMURF1
SNIP1
SOCS1
SOCS6
SOX2
SOX4
SP1
SP3
SPARC
SPRY2
SREK1

SRP54
SSRP1
ST13
ST7L
ST8SIA4
STAT1
STAT3
STAT5A
STMN1
STRN3
SUGT1
SWT1
TAB1
TAL1
TAX1BP1
TCEAL1
TERF2
TERT
TGFB1
TGFBR2
TGOLN2
TIMP2
TLR2
TLR4
TMEM107
TMEM64
TMF1
TMPO
TNFAIP3
TNFRSF13C
TNFSF10
TOP1
TOX
TP53
TPPP
TRAF5
TRAF6
TRIM63
TRIM72
TSC1
TTC4
TTC9C
TTN
TUG1

TWIST1
UBR3
UHRF1
ULK2
UNC13A
USP34
USP48
USP9X
UVRAG
VAV3
VEGFA
VMP1
WDFY2
WDR4
WEE1
WNK3
WNT1
WNT7A
WWP1
XBP1
XIAP
XIST
ZBTB2
ZC3H4
ZEB1
ZEB2
ZFHX4
ZFX
ZIC5
ZKSCAN1
ZNF154
ZNF367
ZNF772
ZNRF3
