# Supplementary material for: Circulating miRNA Profiling in Plasma Samples of Ovarian Cancer Patients
Source: Int J Mol Sci. 2019 Sep 13;20(18):4533. doi: 10.3390/ijms20184533 (PMC6769773; doi:10.3390/ijms20184533)
Supplement: Supplementary file 1 [file ijms-20-04533-s001.zip › Supplementary Table 3.docx]

**Table 3.** The functional annotation of target genes of Gr 1 miRNAs based on their enrichment in specific KEGG pathways.

| \| **Gr. 1 miRNAs KEGG** \| **Gene Count** \| **P Value** \| **Fold Enrichment** \| \| --- \| --- \| --- \| --- \| |  |  |  |
| --- | --- | --- | --- | --- | --- | --- | --- |
| \| hsa05200:Pathways in cancer \| 42 \| 9.6869E-19 \| 5.0700 \| \| --- \| --- \| --- \| --- \| \| hsa04151:PI3K-Akt signaling pathway \| 33 \| 4.0540E-13 \| 4.5378 \| \| hsa05205:Proteoglycans in cancer \| 25 \| 2.1642E-12 \| 5.9301 \| \| hsa04068:FoxO signaling pathway \| 19 \| 2.3841E-10 \| 6.7267 \| \| hsa05230:Central carbon metabolism in cancer \| 14 \| 4.5250E-10 \| 10.3778 \| \| hsa05169:Epstein-Barr virus infection \| 16 \| 2.8310E-8 \| 6.2218 \| \| hsa04066:HIF-1 signaling pathway Hypoxia-inducible factor 1 (HIF-1) \| 14 \| 7.8362E-8 \| 6.9185 \| \| hsa05213:Endometrial cancer \| 11 \| 8.8183E-8 \| 10.0356 \| \| hsa04919:Thyroid hormone signaling pathway \| 15 \| 9.5781E-8 \| 6.1880 \| \| hsa05203:Viral carcinogenesis \| 17 \| 5.1180E-6 \| 3.9341 \| \| hsa04620:Toll-like receptor signaling pathway \| 12 \| 1.1690E-5 \| 5.3707 \| \| hsa04012:ErbB signaling pathway \| 11 \| 1.1935E-5 \| 5.9983 \| \| hsa04150:mTOR signaling pathway \| 9 \| 2.4024E-5 \| 7.3615 \| \| hsa04014:Ras signaling pathway \| 16 \| 6.9494E-5 \| 3.3586 \| \| hsa04931:Insulin resistance \| 11 \| 7.9440E-5 \| 4.8319 \| \| hsa04510:Focal adhesion \| 15 \| 9.5188E-5 \| 3.4544 \| \| hsa04015:Rap1 signaling pathway \| 15 \| 1.1699E-4 \| 3.3886 \| \| hsa04152:AMPK signaling pathway \| 11 \| 2.3561E-4 \| 4.2427 \| \| hsa04110:Cell cycle \| 11 \| 2.5175E-4 \| 4.2085 \| \| hsa04390:Hippo signaling pathway \| 12 \| 3.0203E-4 \| 3.7701 \| \| hsa04350:TGF-beta signaling pathway \| 9 \| 3.4560E-4 \| 5.0830 \| \| hsa04115:p53 signaling pathway \| 8 \| 4.6347E-4 \| 5.6646 \| \| hsa04917:Prolactin signaling pathway \| 8 \| 6.6112E-4 \| 5.3455 \| \| hsa04722:Neurotrophin signaling pathway \| 10 \| 8.7443E-4 \| 3.9534 \| \| hsa04071:Sphingolipid signaling pathway \| 10 \| 8.7443E-4 \| 3.9534 \| |  |  |  |

**Table 1.** The functional annotation of target genes of Gr 2 miRNAs based on their enrichment in specific KEGG pathways.

| \| **Gr. 2 miRNAs KEGG** \| **Gene Count** \| **P Value** \| **Fold Enrichment** \| \| --- \| --- \| --- \| --- \| |  |  |  |
| --- | --- | --- | --- | --- | --- | --- | --- |
| \| hsa05200:Pathways in cancer \| 26 \| 2.5067E-11 \| 4.8935 \| \| --- \| --- \| --- \| --- \| \| hsa04151:PI3K-Akt signaling pathway \| 20 \| 9.8090E-8 \| 4.2879 \| \| hsa05205:Proteoglycans in cancer \| 12 \| 6.5242E-5 \| 4.4380 \| \| hsa04068:FoxO signaling pathway \| 10 \| 6.8271E-5 \| 5.5199 \| \| hsa05206:MicroRNAs in cancer \| 14 \| 9.8261E-5 \| 3.6207 \| \| hsa04931:Insulin resistance \| 8 \| 5.6630E-4 \| 5.4790 \| \| hsa04152:AMPK signaling pathway \| 8 \| 0.0012 \| 4.8109 \| \| hsa05230:Central carbon metabolism in cancer \| 6 \| 0.0015 \| 6.9344 \| \| hsa04920:Adipocytokine signaling pathway \| 6 \| 0.0023 \| 6.3400 \| \| hsa04520:Adherens junction \| 6 \| 0.0024 \| 6.2507 \| \| hsa05213:Endometrial cancer \| 5 \| 0.0049 \| 7.1122 \| \| hsa04350:TGF-beta signaling pathway \| 6 \| 0.0051 \| 5.2834 \| \| hsa05202:Transcriptional misregulation in cancer \| 8 \| 0.0068 \| 3.5433 \| \| hsa04210:Apoptosis \| 5 \| 0.0092 \| 5.9651 \| \| hsa04922:Glucagon signaling pathway \| 6 \| 0.0102 \| 4.4828 \| \| hsa04390:Hippo signaling pathway \| 7 \| 0.0153 \| 3.4289 \| \| hsa04064:NF-kappa B signaling pathway \| 5 \| 0.0287 \| 4.2510 \| \| hsa04621:NOD-like receptor signaling pathway \| 4 \| 0.0385 \| 5.2834 \| \| hsa04550:Signaling pathways regulating pluripotency of stem cells \| 6 \| 0.0390 \| 3.1700 \| \| hsa04066:HIF-1 signaling pathway \| 5 \| 0.0392 \| 3.8524 \| \| hsa04915:Estrogen signaling pathway \| 5 \| 0.0431 \| 3.73575 \| \| hsa04010:MAPK signaling pathway \| 8 \| 0.0520 \| 2.3389 \| \| hsa04510:Focal adhesion \| 7 \| 0.0571 \| 2.5134 \| \| hsa04115:p53 signaling pathway \| 4 \| 0.0600 \| 4.4159 \| \| hsa04722:Neurotrophin signaling pathway \| 5 \| 0.0764 \| 3.0819 \| |  |  |  |

**Table 1.** The functional annotation of target genes of Gr 3 miRNAs based on their enrichment in specific KEGG pathways.

| \| **Gr. 3 miRNAs KEGG** \| **Gene Count** \| **P Value** \| **Fold Enrichment** \| \| --- \| --- \| --- \| --- \| |  |  |  |
| --- | --- | --- | --- | --- | --- | --- | --- |
| \| hsa05206:MicroRNAs in cancer \| 23 \| 1.5576E-11 \| 5.8851 \| \| --- \| --- \| --- \| --- \| \| hsa05200:Pathways in cancer \| 26 \| 3.2507E-11 \| 4.84145 \| \| hsa04151:PI3K-Akt signaling pathway \| 24 \| 9.2667E-11 \| 5.0908 \| \| hsa04068:FoxO signaling pathway \| 14 \| 2.23032E-8 \| 7.6457 \| \| hsa05205:Proteoglycans in cancer \| 15 \| 3.8433E-7 \| 5.4885 \| \| hsa04066:HIF-1 signaling pathway \| 11 \| 5.4183E-7 \| 8.3853 \| \| hsa04110:Cell cycle \| 12 \| 7.3022E-7 \| 7.0820 \| \| hsa04550:Signaling pathways regulating pluripotency of stem cells \| 12 \| 2.4523E-6 \| 6.2726 \| \| hsa05202:Transcriptional misregulation in cancer \| 12 \| 1.3523E-5 \| 5.2585 \| \| hsa04630:Jak-STAT signaling pathway \| 11 \| 2.3004E-5 \| 5.5516 \| \| hsa04910:Insulin signaling pathway \| 10 \| 9.3449E-5 \| 5.3029 \| \| hsa04350:TGF-beta signaling pathway \| 8 \| 1.2771E-4 \| 6.9696 \| \| hsa05321:Inflammatory bowel disease (IBD) \| 7 \| 2.0712E-4 \| 8.0041 \| \| hsa04152:AMPK signaling pathway \| 9 \| 2.3780E-4 \| 5.3546 \| \| hsa04115:p53 signaling pathway \| 7 \| 2.6688E-4 \| 7.6457 \| \| hsa04917:Prolactin signaling pathway \| 7 \| 3.6677E-4 \| 7.2150 \| \| hsa04660:T cell receptor signaling pathway \| 8 \| 3.7883E-4 \| 5.8544 \| \| hsa04510:Focal adhesion \| 11 \| 4.3102E-4 \| 3.9077 \| \| hsa04931:Insulin resistance \| 8 \| 6.0459E-4 \| 5.4208 \| \| hsa04062:Chemokine signaling pathway \| 10 \| 8.6489E-4 \| 3.9344 \| \| hsa04621:NOD-like receptor signaling pathway \| 6 \| 8.9869E-4 \| 7.84080 \| \| hsa04150:mTOR signaling pathway \| 6 \| 0.0010 \| 7.5704 \| \| hsa04914:Progesterone-mediated oocyte maturation \| 7 \| 0.0010 \| 5.8881 \| \| hsa04012:ErbB signaling pathway \| 7 \| 0.0011 \| 5.8881 \| \| hsa05230:Central carbon metabolism in cancer \| 5 \| 0.0107 \| 5.7172 \| |  |  |  |

**Table 1.** The GO-based functional annotation of target genes of Gr 1 miRNAs based on their enrichment in specific GO terms.

| \| **Gr. 1 miRNAs GO Biol Proc** \| **Gene Count** \| **P Value** \| **Fold Enrichment** \| \| --- \| --- \| --- \| --- \| |  |  |  |
| --- | --- | --- | --- | --- | --- | --- | --- |
| \| GO:0048015~phosphatidylinositol-mediated signaling \| 13 \| 1.3427E-8 \| 9.3608 \| \| --- \| --- \| --- \| --- \| \| GO:2000352~negative regulation of endothelial cell apoptotic process \| 7 \| 1.3620E-6 \| 19.0818 \| \| GO:0043552~positive regulation of phosphatidylinositol 3-kinase activity \| 7 \| 2.5763E-6 \| 17.2351 \| \| GO:0007179~transforming growth factor beta receptor signaling pathway \| 10 \| 3.1511E-6 \| 8.2964 \| \| GO:1902042~negative regulation of extrinsic apoptotic signaling pathway via death domain receptors \| 7 \| 3.7920E-6 \| 16.1906 \| \| GO:0014066~regulation of phosphatidylinositol 3-kinase signaling \| 9 \| 7.8861E-6 \| 8.8069 \| \| GO:0051592~response to calcium ion \| 8 \| 9.9686E-6 \| 10.5278 \| \| GO:0007249~I-kappaB kinase/NF-kappaB signaling \| 8 \| 1.2524E-5 \| 10.1769 \| \| GO:0046854~phosphatidylinositol phosphorylation \| 9 \| 3.1322E-5 \| 7.3079 \| \| GO:0018108~peptidyl-tyrosine phosphorylation \| 11 \| 3.2817E-5 \| 5.4875 \| \| GO:0000165~MAPK cascade \| 14 \| 4.1488E-5 \| 4.0785 \| \| GO:0051092~positive regulation of NF-kappaB transcription factor activity \| 10 \| 6.2259E-5 \| 5.7388 \| \| GO:0002755~MyD88-dependent toll-like receptor signaling pathway \| 6 \| 6.3526E-5 \| 13.8776 \| \| GO:0050731~positive regulation of peptidyl-tyrosine phosphorylation \| 8 \| 9.6758E-5 \| 7.4465 \| \| GO:0070423~nucleotide-binding oligomerization domain containing signaling pathway \| 5 \| 2.8714E-4 \| 15.2654 \| \| GO:0007254~JNK cascade \| 6 \| 4.3093E-4 \| 9.3461 \| \| GO:0060389~pathway-restricted SMAD protein phosphorylation \| 4 \| 5.6820E-4 \| 23.4853 \| \| GO:0038095~Fc-epsilon receptor signaling pathway \| 9 \| 0.0023 \| 3.8592 \| \| GO:0030509~BMP signaling pathway \| 6 \| 0.0031 \| 6.0258 \| \| GO:0008286~insulin receptor signaling pathway \| 6 \| 0.0035 \| 5.8713 \| \| GO:0036092~phosphatidylinositol-3-phosphate biosynthetic process \| 5 \| 0.00373 \| 7.78849 \| \| GO:0090277~positive regulation of peptide hormone secretion \| 3 \| 0.0045 \| 28.6227 \| \| GO:0051897~positive regulation of protein kinase B signaling \| 6 \| 0.0048 \| 5.45194 \| \| GO:0031663~lipopolysaccharide-mediated signaling pathway \| 4 \| 0.0082 \| 9.54090 \| \| GO:1900026~positive regulation of substrate adhesion-dependent cell spreading \| 4 \| 0.0082 \| 9.5409 \| |  |  |  |

**Table 1.** The GO-based functional annotation of target genes of Gr 2 miRNAs based on their enrichment in specific GO terms.

| \| **Gr. 2 miRNAs GO Biol Proc** \| **Gene Count** \| **P Value** \| **Fold Enrichment** \| \| --- \| --- \| --- \| --- \| |
| --- | --- | --- | --- | --- |
| \| GO:0048015~phosphatidylinositol-mediated signaling \| 13 \| 1.3427E-8 \| 9.3608 \| \| --- \| --- \| --- \| --- \| \| GO:2000352~negative regulation of endothelial cell apoptotic process \| 7 \| 1.3620E-6 \| 19.0818 \| \| GO:0043552~positive regulation of phosphatidylinositol 3-kinase activity \| 7 \| 2.5763E-6 \| 17.2351 \| \| GO:0007179~transforming growth factor beta receptor signaling pathway \| 10 \| 3.1511E-6 \| 8.2964 \| \| GO:1902042~negative regulation of extrinsic apoptotic signaling pathway via death domain receptors \| 7 \| 3.7920E-6 \| 16.1906 \| \| GO:0014066~regulation of phosphatidylinositol 3-kinase signaling \| 9 \| 7.8861E-6 \| 8.8069 \| \| GO:0051592~response to calcium ion \| 8 \| 9.9686E-6 \| 10.5278 \| \| GO:0007249~I-kappaB kinase/NF-kappaB signaling \| 8 \| 1.2524E-5 \| 10.1769 \| \| GO:0046854~phosphatidylinositol phosphorylation \| 9 \| 3.1322E-5 \| 7.3079 \| \| GO:0018108~peptidyl-tyrosine phosphorylation \| 11 \| 3.2817E-5 \| 5.4875 \| \| GO:0000165~MAPK cascade \| 14 \| 4.1488E-5 \| 4.0785 \| \| GO:0051092~positive regulation of NF-kappaB transcription factor activity \| 10 \| 6.2259E-5 \| 5.7388 \| \| GO:0002755~MyD88-dependent toll-like receptor signaling pathway \| 6 \| 6.3526E-5 \| 13.8776 \| \| GO:0050731~positive regulation of peptidyl-tyrosine phosphorylation \| 8 \| 9.6758E-5 \| 7.4465 \| \| GO:0070423~nucleotide-binding oligomerization domain containing signaling pathway \| 5 \| 2.8714E-4 \| 15.2654 \| \| GO:0007254~JNK cascade \| 6 \| 4.3093E-4 \| 9.3461 \| \| GO:0060389~pathway-restricted SMAD protein phosphorylation \| 4 \| 5.6820E-4 \| 23.4853 \| \| GO:0038095~Fc-epsilon receptor signaling pathway \| 9 \| 0.0023 \| 3.8592 \| \| GO:0030509~BMP signaling pathway \| 6 \| 0.0031 \| 6.0258 \| \| GO:0008286~insulin receptor signaling pathway \| 6 \| 0.0035 \| 5.8713 \| \| GO:0036092~phosphatidylinositol-3-phosphate biosynthetic process \| 5 \| 0.00373 \| 7.78849 \| \| GO:0090277~positive regulation of peptide hormone secretion \| 3 \| 0.0045 \| 28.6227 \| \| GO:0051897~positive regulation of protein kinase B signaling \| 6 \| 0.0048 \| 5.45194 \| \| GO:0031663~lipopolysaccharide-mediated signaling pathway \| 4 \| 0.0082 \| 9.54090 \| \| GO:1900026~positive regulation of substrate adhesion-dependent cell spreading \| 4 \| 0.0082 \| 9.5409 \| |

**Table 1.** The GO-based functional annotation of target genes of Gr 3 miRNAs based on their enrichment in specific GO terms.

| \| **Gr. 3 miRNAs GO Biol Proc** \| **Gene Count** \| **P Value** \| **Fold Enrichment** \| \| --- \| --- \| --- \| --- \| |
| --- | --- | --- | --- | --- |
| \| GO:0045944~positive regulation of transcription from RNA polymerase II promoter \| 46 \| 9.1268E-21 \| 9.3608 \| \| --- \| --- \| --- \| --- \| \| GO:0043066~negative regulation of apoptotic process \| 22 \| 6.1513E-10 \| 19.0818 \| \| GO:0010628~positive regulation of gene expression \| 16 \| 1.2008E-8 \| 17.2351 \| \| GO:0008284~positive regulation of cell proliferation \| 19 \| 1.6961E-7 \| 8.2964 \| \| GO:0071456~cellular response to hypoxia \| 9 \| 2.1828E-6 \| 16.1906 \| \| GO:0008283~cell proliferation \| 15 \| 4.8407E-6 \| 8.8069 \| \| GO:0032869~cellular response to insulin stimulus \| 7 \| 6.2288E-5 \| 10.5278 \| \| GO:0006260~DNA replication \| 9 \| 7.2942E-5 \| 10.1769 \| \| GO:0050731~positive regulation of peptidyl-tyrosine phosphorylation \| 7 \| 8.8709E-5 \| 7.3079 \| \| GO:0045892~negative regulation of transcription, DNA-templated \| 15 \| 1.4491E-4 \| 5.4875 \| \| GO:0070301~cellular response to hydrogen peroxide \| 6 \| 1.4852E-4 \| 4.0785 \| \| GO:0050679~positive regulation of epithelial cell proliferation \| 6 \| 1.8964E-4 \| 5.7388 \| \| GO:0000082~G1/S transition of mitotic cell cycle \| 7 \| 2.9503E-4 \| 13.8776 \| \| GO:0042517~positive regulation of tyrosine phosphorylation of Stat3 protein \| 5 \| 3.4756E-4 \| 7.4465 \| \| GO:0048015~phosphatidylinositol-mediated signaling \| 7 \| 3.6309E-4 \| 15.2654 \| \| GO:0033138~positive regulation of peptidyl-serine phosphorylation \| 6 \| 3.9143E-4 \| 9.3461 \| \| GO:0042523~positive regulation of tyrosine phosphorylation of Stat5 protein \| 4 \| 4.2497E-4 \| 23.4853 \| \| GO:0010595~positive regulation of endothelial cell migration \| 5 \| 7.2730E-4 \| 3.8592 \| \| GO:0008285~negative regulation of cell proliferation \| 12 \| 8.22971E-4 \| 6.0258 \| \| GO:1901796~regulation of signal transduction by p53 class mediator \| 7 \| 8.34377E-4 \| 5.8713 \| \| GO:0051726~regulation of cell cycle \| 7 \| 8.34377E-4 \| 7.78849 \| \| GO:0051897~positive regulation of protein kinase B signaling \| 6 \| 9.0414E-4 \| 28.6227 \| \| GO:0000715~nucleotide-excision repair, DNA damage recognition \| 4 \| 0.0010 \| 5.45194 \| \| GO:0045727~positive regulation of translation \| 5 \| 0.0012 \| 9.54090 \| \| GO:0032355~response to estradiol \| 6 \| 0.0012 \| 9.5409 \| |
