# Supplementary material for: Circulating miRNA Profiling in Plasma Samples of Ovarian Cancer Patients
Source: Int J Mol Sci. 2019 Sep 13;20(18):4533. doi: 10.3390/ijms20184533 (PMC6769773; doi:10.3390/ijms20184533)
Supplement: Supplementary file 1 [file ijms-20-04533-s001.zip › Supplemetary Figure 1a-c.pdf]

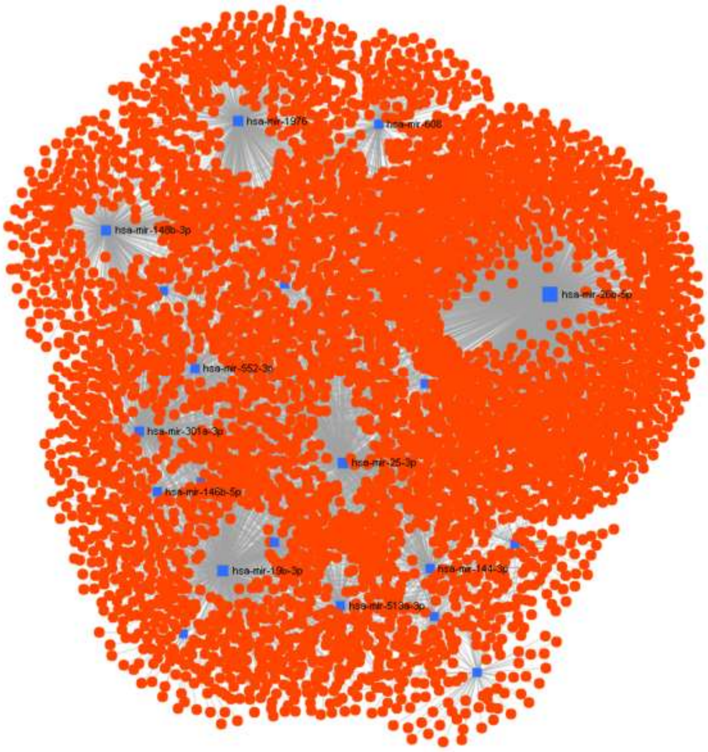

Figure 1a. The complete miRNA-taget proteins interaction network of Group 1 miRNAs.

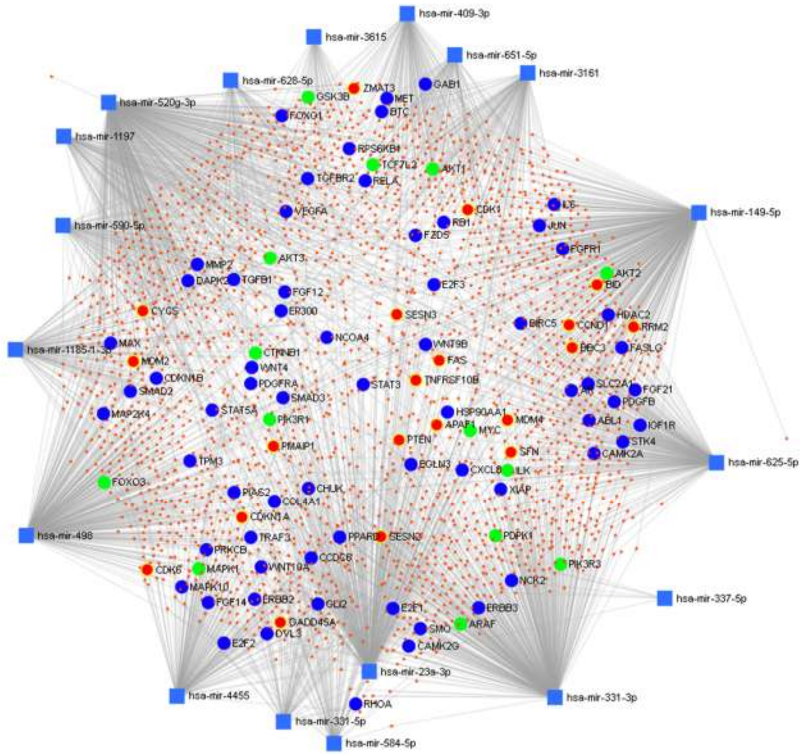

Figure 1b. The complete miRNA-taget proteins interaction network of Group 2 miRNAs. The colored hubes are proteins known to play a role in tumorogenezis.

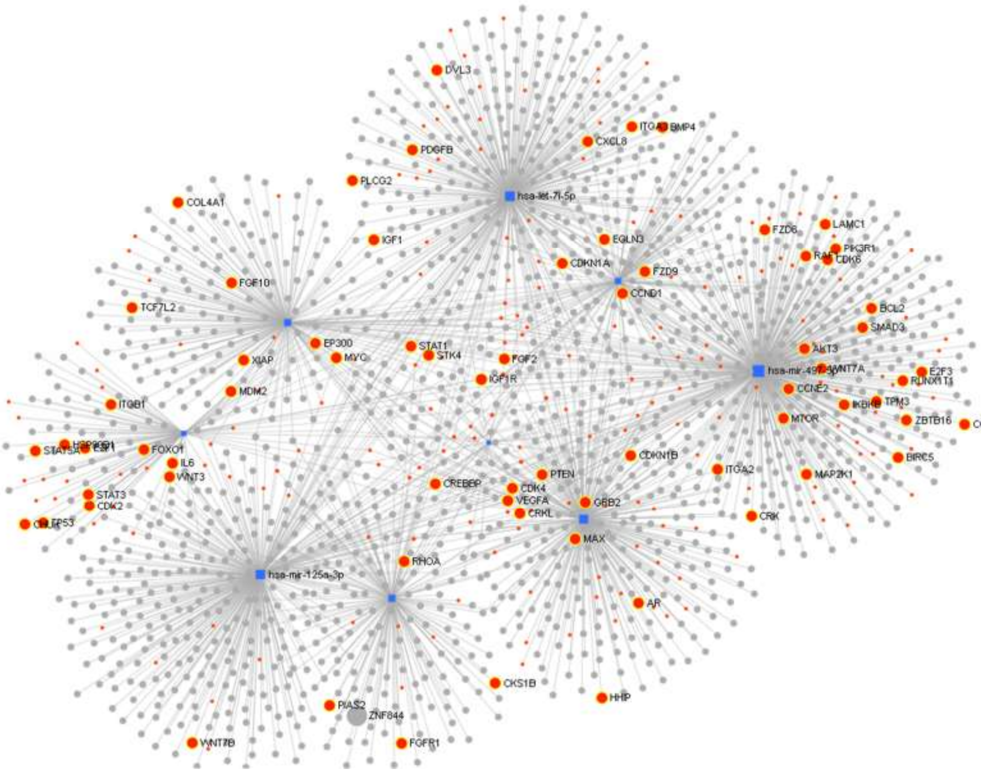

Figure 1c. The complete miRNA-taget proteins interaction network of Group 3 miRNAs. The colored hubes are proteins known to play a role in tumorogenezis.
